# Orbital-anisotropic electronic structure in the nonmagnetic state of BaFe_2_(As_1−*x*_P_*x*_)_2_ superconductors

**DOI:** 10.1038/s41598-018-20332-1

**Published:** 2018-02-01

**Authors:** T. Sonobe, T. Shimojima, A. Nakamura, M. Nakajima, S. Uchida, K. Kihou, C. H. Lee, A. Iyo, H. Eisaki, K. Ohgushi, K. Ishizaka

**Affiliations:** 1 Quantum-Phase Electronics Center (QPEC) and Department of Applied Physics, The University of Tokyo, Bunkyo, Tokyo, 113-8656 Japan; 2 RIKEN Center for Emergent Matter Science (CEMS), Wako, 351-0198 Japan; 3 Department of Physics, Osaka University, Toyonaka, Osaka, 560-8531 Japan; 4 Department of Physics, The University of Tokyo, Bunkyo, Tokyo, 113-0033 Japan; 5 National Institute of Advanced Industrial Science and Technology, Tsukuba, 305-8568 Japan; 6 Department of Physics, Tohoku University, Sendai, Miyagi 980-8578 Japan

## Abstract

High-temperature superconductivity in iron-pnictides/chalcogenides arises in balance with several electronic and lattice instabilities. Beside the antiferromagnetic order, the orbital anisotropy between Fe 3*d*_*xz*_ and 3*d*_*yz*_ occurs near the orthorhombic structural transition in several parent compounds. However, the extent of the survival of orbital anisotropy against the ion-substitution remains to be established. Here we report the composition (*x*) and temperature (*T*) dependences of the orbital anisotropy in the electronic structure of a BaFe_2_(As_1−*x*_P_*x*_)_2_ system by using angle-resolved photoemission spectroscopy. In the low-*x* regime, the orbital anisotropy starts to evolve on cooling from high temperatures above both antiferromagnetic and orthorhombic transitions. By increasing *x*, it is gradually suppressed and survives in the optimally doped regime. We find that the in-plane orbital anisotropy persists in a large area of the nonmagnetic phase, including the superconducting dome. These results suggest that the rotational symmetry-broken electronic state acts as the stage for superconductivity in BaFe_2_(As_1−*x*_P_*x*_)_2_.

## Introduction

High-transition temperature (*T*_c_) superconductivity in iron-pnictides/chalcogenides^1^ emerges from the multi-band electronic structure near the Fermi level (*E*_F_) composed of the five Fe 3*d* orbitals. Most of the parent compounds exhibit an antiferromagnetic (AF) order at *T*_N_ and tetragonal-to-orthorhombic structural transitions just at or slightly above *T*_N_ (*T*_s_). In addition, the lifting of degeneracy in Fe 3*d*_*xz*_/*3d*_*yz*_ (*xz*/*yz*) orbitals, the so-called orbital anisotropy^2–4^ was observed by angle-resolved photoemission spectroscopy (ARPES), for various materials, such as the BaFe_2_As_2_ family^5–7^, NaFeAs^8,9^, and FeSe^10,11^. The orbital anisotropy is a possible explanation for the origin of the electronic nematicity^8,10–13^ which has been intensively studied in terms of magnetic origin^14,15^. It is theoretically suggested that the orbital fluctuation evolves near the orbital-ordered state^16,17^. This results in a sign-preserved *s*_++_-wave superconductivity, whereas a sign-changing *s*_±_-wave superconductivity is induced by the spin fluctuation^18,19^. The nature of the orbital order, in particular, the extent of the persistence of the orbital anisotropy against the ion-substitution, can help understand the mechanism of high-*T*_c_ superconducting (SC) transition in iron-based materials.

The persistence of the orbital-anisotropic state, however, has only been investigated for some shallow-doped Ba(Fe_1-*x*_Co_*x*_)_2_As_2_ materials^6,20^. For an accurate tracking of the intrinsic orbital anisotropy beyond the SC dome, both materials and methods need to be selected carefully. The application of ARPES on strain-free crystals enables the avoidance of an extrinsic anisotropy which increases the onset *T* of the orbital anisotropy (*T*_o_)^21^. In addition, the isovalent-ion substituted system is the most suitable for the thorough investigation of the degeneracy lifting in the *xz*/*yz* orbitals, without the influence of the chemical potential shift induced by carrier doping. Thus, we used ARPES on strain-free BaFe_2_(As_1−*x*_P_*x*_)_2_ (AsP122) crystals with 0.00 ≤ *x* ≤ 0.87 for the study of the *x–T* region showing an orbital-anisotropic electronic structure.

## Results

### The *x*-dependence of the *E-k* images around the X/Y point

**Figure 1:**
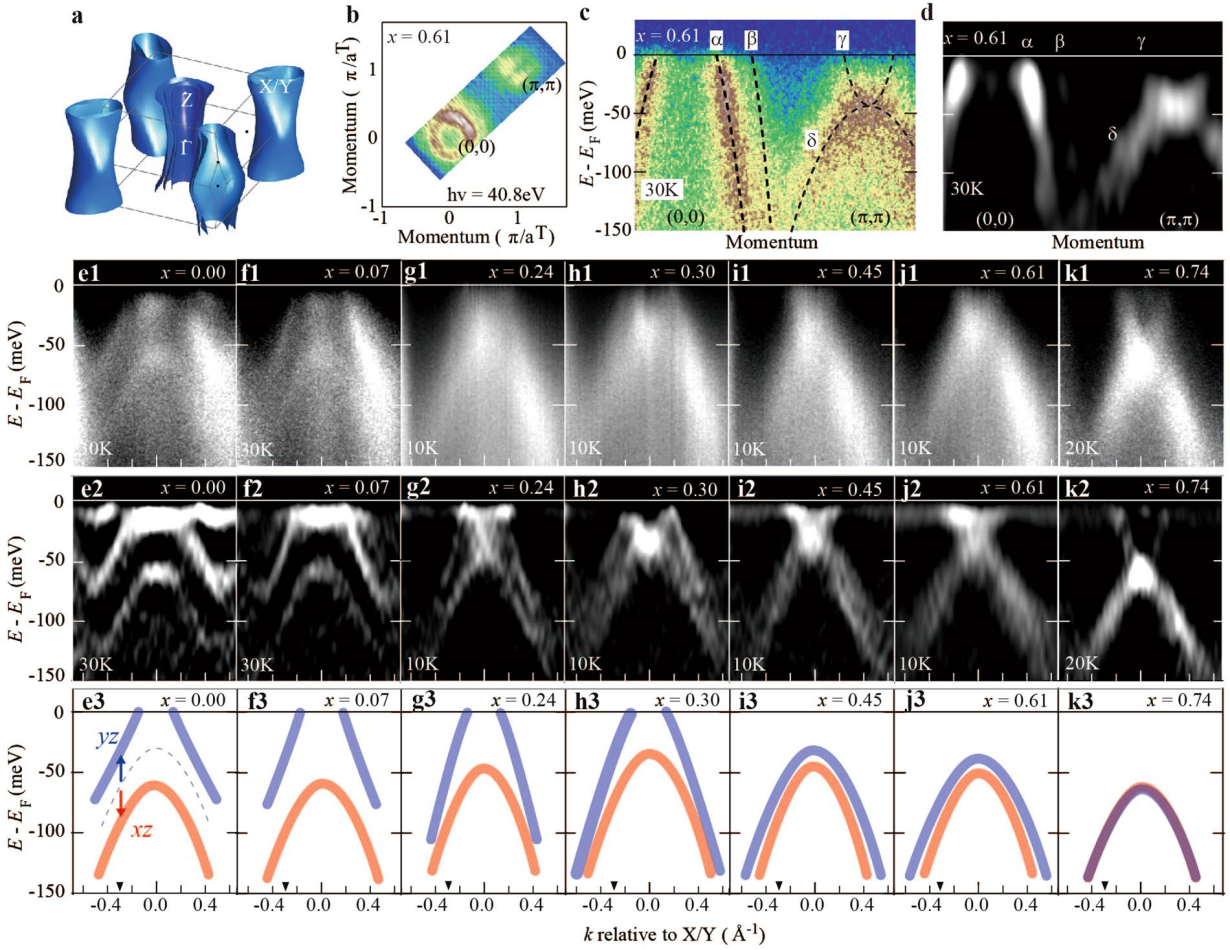
(**a**) First-principles band calculations of FSs for *x* = 0.00. **(b)** FS mapping at *x* = 0.61 by *hν* = 40.8 eV. *a*^*T*^ represents the inplane lattice parameter of the tetragonal structure. **(c**,**d)** *E*-*k* image and its second *E* derivative image for *x* = 0.61 along (0,0) – (π,π) direction. Two electron bands crossing *E*_F_ around (π,π) is not separately detected in this experimental condition. (**e1**–**k1**) *E*-*k* images at the BZ corner on twinned crystals of 0.00 ≤ *x* ≤ 0.74. (**e2**–**k2**) The second *E* derivative *E-k* images of 0.00 ≤ *x* ≤ 0.74. (**e3**–**k3**) Schematic band dispersions obtained from (e2) to (k2). Blue and red curves correspond to the band dispersions of *yz* and *xz* orbitals, respectively. Broken curve in (e3) shows the degenerate *xz/yz* band at higher *T*.

The electronic structure of BaFe_2_As_2_ is highly two-dimensional as indicated by the calculated Fermi surfaces (FSs) in Fig. 1a. Figure 1b shows the FS image for *x* = 0.61 from the ARPES with *hν* = 40.8 eV. The energy–momentum (*E*–*k*) image along (0,0) and (π,π) and the image of its second *E* derivative are shown in Fig. 1c and d, respectively. They exhibit two hole bands at the Brillouin zone (BZ) center (*α* and *β* bands), and an electron band and a hole band at the BZ corner (*γ* and *δ* bands). According to the band calculation, the *δ* band reaching the *γ* band at 50 meV below *E*_F_ is composed of *xz/yz* orbital characters. Previous ARPES on BaFe_2_As_2_ demonstrated that degeneracy lifting in the *xz*/*yz* orbitals appears in the *δ* band in the orthorhombic AF state^6^.

Here, we focus on the *δ* band at the BZ corner at low *T*, for the strain-free (twinned) crystals, to determine the orbital anisotropy *Φ*_o_ = *E*_*yz*_ − *E*_*xz*_. Figures 1e1–k1 and e2–k2 show the *E*–*k* images and their second *E* derivative around the BZ corner for 0.00 ≤ *x* ≤ 0.74, respectively. For *x* = 0.00, we observed a pair of hole bands with similar dispersions as indicated by the red and blue curves in Fig. 1e3. Previous ARPES on de-twinned BaFe_2_As_2_ crystals confirmed the upward shift of the *yz* hole band and the downward shift of the *xz* hole band, appearing at different BZ corners^6^ (*X* and *Y* points). In the case of twinned crystals, the *δ* bands composed of *yz* and *xz* orbitals at the *X* and *Y* points overlap in the *E–k* image at the BZ corner. Thus, the observation of the pair of hole bands in the orthorhombic AF state can be interpreted as degeneracy lifting in the *xz*/*yz* orbitals, i.e. in-plane orbital anisotropy.

### The *x*-dependence of the second *E* derivative of the energy distribution curves

**Figure 2:**
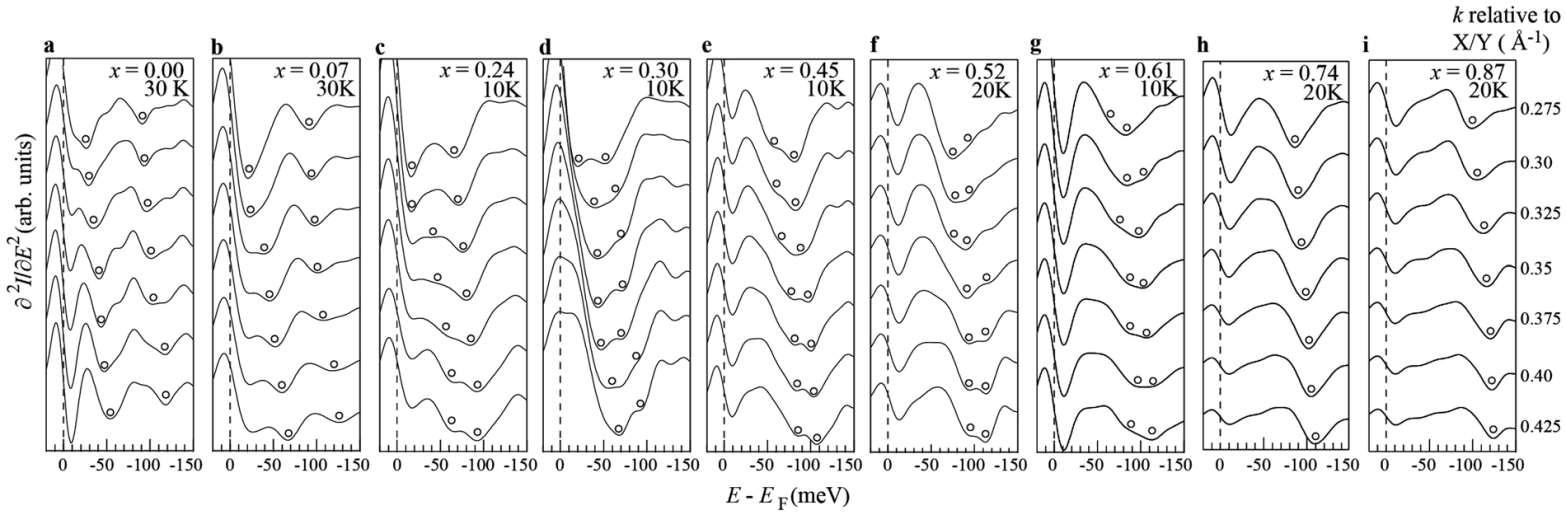
(**a**–**i**) *x* dependence of second *E* derivative EDCs in an interval of 0.025 Å^−1^ near *X*/*Y* point at 30 K for *x* = 0.00 and 0.07, 20 K for *x* = 0.52, 0.74 and 0.87, 10 K for *x* = 0.24, 0.30, 0.45 and 0.61, respectively. Black open circles represent dip positions in the spectra corresponding to the energy position of the *δ* hole bands.

The schematics of the band dispersions around *X/Y* points shown in Fig. 1e3–1k3 were obtained from the spectral dips of the second *E* derivative of the energy distribution curves (EDCs), which represents the energy levels of the *δ* band at each *k*. Figure 2a–i show the second *E* derivative of the EDCs near the *X*/*Y* point in an interval of 0.025 Å^−1^ at 30 K for *x* = 0.00 and 0.07, at 20 K for *x* = 0.52, 0.74, 0.87, and at 10 K for *x* = 0.30, 0.45, and 0.61, respectively. The systematic parallel shift of the double dip feature as a function of *k* suggests a clear degeneracy lifting in the *xz/yz* orbitals in the *δ* bands for 0.0 ≤ *x* ≤ 0.30. For higher *x* values, the orbital anisotropy becomes unclear and, eventually, a single *δ* band was observed for *x* = 0.74 and 0.87 down to 20 K. These observations indicate that an orbital anisotropy persists in the optimally doped regime and possibly higher doping level where static orthorhombicity and antiferromagnetism no longer exist.

### *E-k* images around X and Y points of the detwinned single crystals for *x* = 0.3

**Figure 3:**
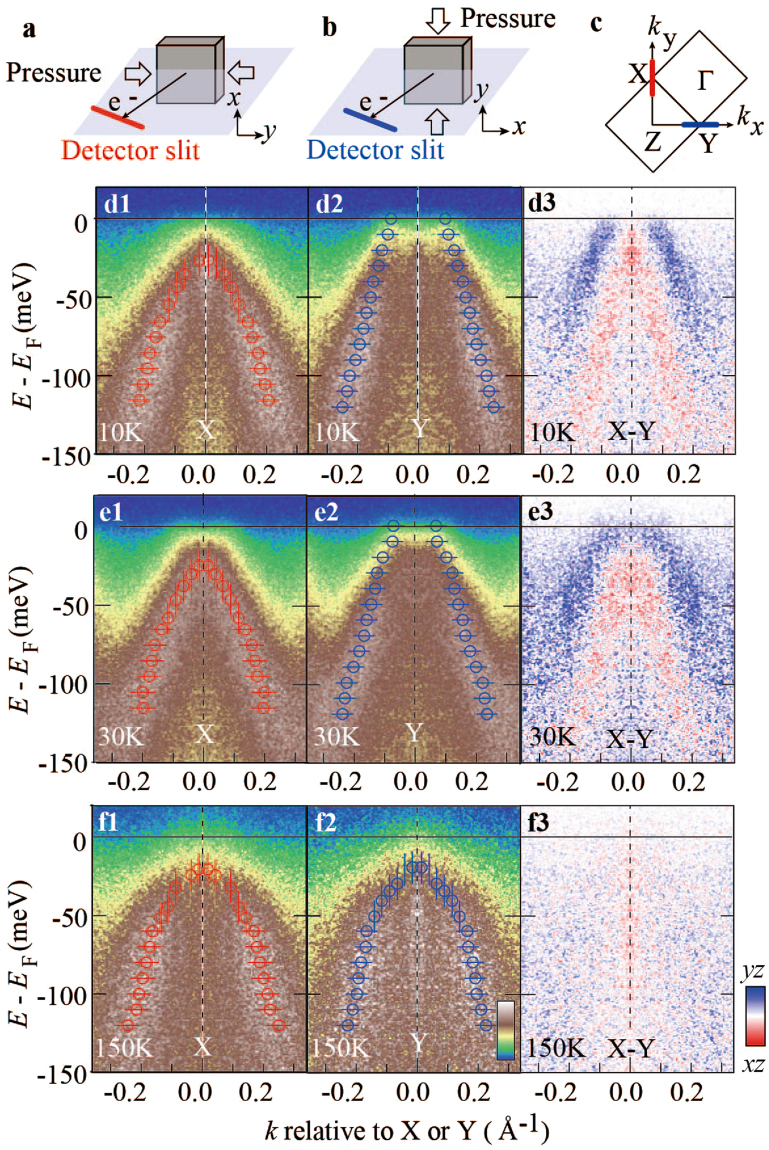
**(a,b)** Geometry 1 and 2 for the ARPES on de-twinned crystals, respectively. **(c)** Momentum cut corresponding to the Geometry 1 (2) as indicated by a red (blue) line. Photons of *hν* = 40.8 eV probe the BZ center near the Γ plane and the BZ corner near the *Z* plane, respectively. (**d1**,**d2**) ARPES images of de-twinned BaFe_2_(As_0.7_P_0.3_)_2_ at 12 K (*T* < *T*_c_) by around *X* along *k*_*y*_ and *Y* along *k*_*x*_, respectively. The peak positions from EDCs and MDCs of ARPES images are superimposed by red (*xz*) and blue (*yz*) markers, respectively. (**d3**) Differences between APRES images around *X*(||*k*_*y*_) and *Y*(||*k*_*x*_) at 12 K. Red and blue intensities represent *δ* bands of *xz* and *yz* orbitals, respectively. (**e1–e3)** Similar to (d1-d3) recorded at 30 K (*T* ∼ *T*_c_). (**f1–f3**) Similar to (d1–d3) recorded at 150 K (*T* ≫ *T*_c_).

It is worth mentioning that the signature of orbital anisotropy was found even in the tetragonal SC state (Fig. 1h3 and i3). To investigate the degeneracy lifting in the *xz*/*yz* orbitals in the SC state, we performed ARPES on the de-twinned crystals at *x* = 0.30. As schematically shown in Fig. 3a and b, a uniaxial pressure was applied to the single crystals along the [1 1 0] direction in the tetragonal notation. This direction is denoted by the *y*-axis, corresponding to the shorter *b*-axis in the orthorhombic notation. The *x*-axis corresponds to the *a*-axis in the orthorhombic notation. In the ARPES experiments, the crystals were rotated around [0 0 1] *in situ* by 90°, for the separate detection of the *δ* bands along the *k*_*x*_*-* and *k*_*y*_-directions (Fig. 3c).

Figure 3d1 and d2 show the band dispersions symmetrised with respect to the *X* and *Y* points, respectively, obtained for the SC state at *x* = 0.30. The peak positions of the EDCs and momentum distribution curves (MDCs), plotted in Fig. 3d1 and d2, indicate that the *δ* band attains higher energy at *Y* (||*k*_*x*_), thus confirming the existence of orbital anisotropy in the SC state. The first-principles band calculation for BaFe_2_As_2_ indicates that the *δ* band at *X* (||*k*_*y*_) [*Y* (||*k*_*x*_)] is composed of *xz* (*yz*) orbital; these orbitals are marked with red (blue) in Fig. 3d1 and d2. The orbital anisotropy, *Φ*_o_ = *E*_*yz*_*−E*_*xz*_, is determined to be ~30 meV at a momentum cut of ~0.3 Å^−1^ away from the *X*/*Y* point, which is comparable to that of the twinned crystals [Fig. 1h3]. These results indicate that the lifting of the *xz/yz* orbital degeneracy in the SC state is not quantitatively affected by the de-twinning procedure. A similar ARPES measurement at 30 K (*T* ≈ *T*_c_) demonstrates a comparable magnitude of orbital non-equivalency, suggesting that the superconductivity onset has negligible effect on it (Fig. 3e1 and e2). At 150 K (*T* ≫ *T*_c_), the *δ* bands composed of *xz* and *yz* orbitals become equivalent (Fig. 3f1 and f2), thus recovering the C_4_ symmetry of the tetragonal lattice in the high *T* region. To emphasise the separation between the *xz* and *yz* orbital bands, we present images showing the difference of intensities between the *E*–*k* image at *X* (||*k*_*y*_) and that at *Y* (||*k*_*x*_), recorded at 12 K (Fig. 3d3), 30 K (Fig. 3e3), and 150 K (Fig. 3f3).

### Dependence of the orbital anisotropy on *x* and *T*

**Figure 4:**
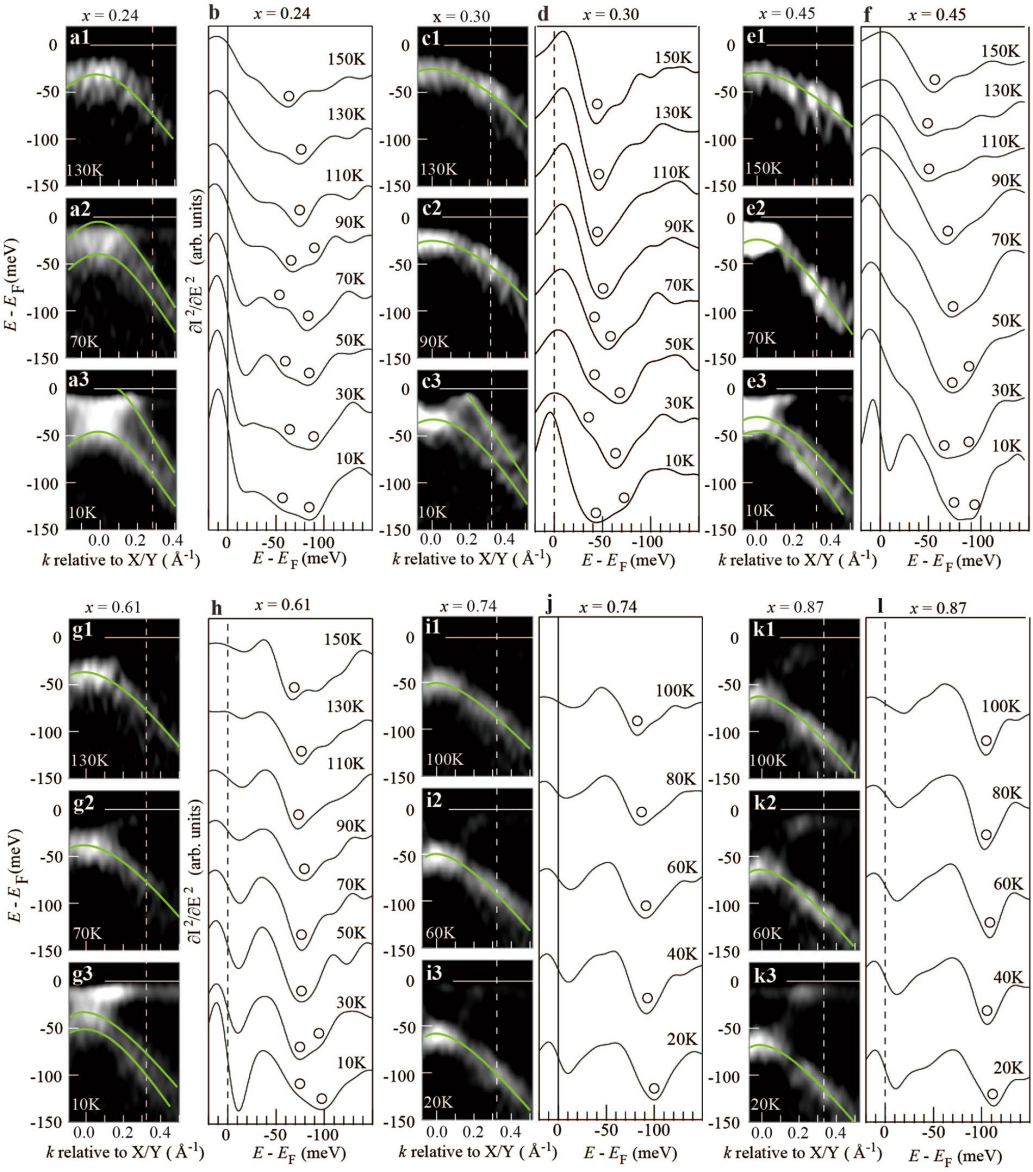
(**a1-a3**) Second *E* derivative of the *E-k* images for *x* = 0.24 taken at 130 K, 70 K and 10 K, respectively. Green curves are the guides to the eyes. **(b)** *T* dependence of the second *E* derivative EDCs of twinned samples of *x* = 0.24 taken at the momentum indicated by the broken lines in (a1-a3). Black open circles represent the spectral dips corresponding to the energy position of *δ* hole bands. **(c1-c3)** The same as (a1-a3) but for *x* = 0.30. **(d)** The same as (b) but for *x* = 0.30. **(e1–e3)** The same as (a1–a3) but for *x* = 0.45. **(f)** The same as (b) but for *x* = 0.45. **(g1–g3)** The same as (a1-a3) but for *x* = 0.61. **(h)**, The same as (b) but for *x* = 0.61. **(i1-i3)** The same as (a1-a3) but for *x* = 0.74. **(j)** The same as (b) but for *x* = 0.74. **(k1–k3)** The same as (a1-a3) but for *x* = 0.87. **(l)** The same as (b) but for *x* = 0.87.

**Figure 5:**
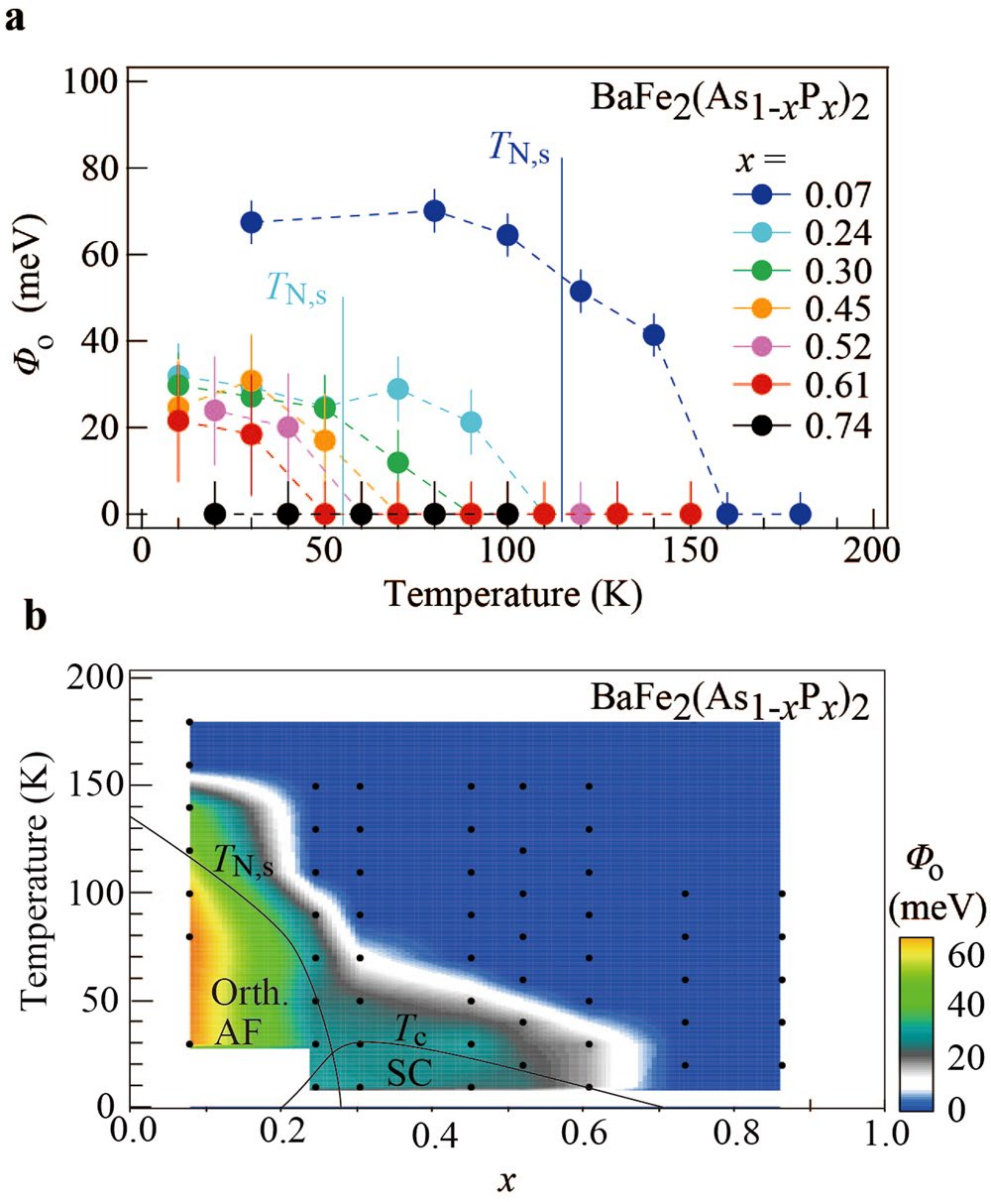
**(a)** Summary of *x* and *T* dependence of the orbital anisotropic parameter *Φ*_o_ = *E*_*yz*_ − *E*_*xz*_ estimated from the second *E* derivative EDCs. **(b)** Contour plot of *Φ*_o_ in the *x*-*T* phase diagram of AsP122 system. The measurement points were indicated by black dots.

To determine the orbital-anisotropic region in the phase diagram, we performed ARPES on an AsP122 system for a wide range of *x* and *T*. Twinned crystals were used as the de-twinning procedure may extrinsically increase *T*_o_^21^. Figures 4a1–a3 show *E–k* images around the *X*/*Y* point for *x* = 0.24 obtained at 130 K, 70 K, and 10 K, respectively. While a single *δ* band was observed at 130 K, it split into two at 70 K and 10 K, as indicated by the green curves. To see the *T*-dependence in detail, Fig. 4b shows the second *E* derivative of the EDCs at the momentum indicated by the dotted white lines in Fig. 4a1–a3, plotted for several *T* values. As can be seen in Fig. 4b, a single dip gradually splits into two with the decrease of *T*, indicating the evolution of the orbital anisotropy. The *T* value where the two-dip structure appears, corresponding to *T*_*o*_, was determined to be ∼110 K for *x* = 0.24. Similarly, *T*_o_ was determined to be ∼90 K for *x* = 0.30, ∼70 K for *x* = 0.45, and ∼50 K for *x* = 0.61, respectively (Fig. 4d,f, and h). It should be noted that the error bars of *T*_o_ are particularly large for 0.45 ≤ *x* ≤ 0.61 as the noise level is in the range of the intrinsic signal. At *x* = 0.74 and 0.87, down to 20 K the double-dip feature can no longer be observed, as shown in Fig. 4j and l. These findings are summarised in Fig. 5a and b. (In this work, we find that the relation of *T*_s_ < *T*_o_ holds in both as-grown and annealed crystals: The annealed crystals are considered to be of high purity and more homogeneous than the as-grown samples. The present result confirms that the inhomogeneity and/or fluctuations in the sample quality have no serious effect on the determination of *T*_o_ in the present ARPES).

## Discussion

As can be seen in Fig. 5b, *T*_o_ and *Φ*_o_ of the orbital anisotropy are monotonically suppressed toward the high-*x* region. Considering that both orthorhombic and AF phases show a similar sudden drop immediately below *x* ≈ 0.30, the orbital anisotropy seems to be decoupled from these two orders. Nevertheless, the *x*–*T* area where the orbital anisotropy appears is nearly equivalent to the electronic nematic region above *T*_s_ and *T*_N_, determined by the torque magnetometry and X-ray diffraction (XRD) measurements^22^. This coincidence suggests that the loss of rotational symmetry in the lattice and magnetism cooperatively evolve with the orbital anisotropy from the high-*T* region. Theoretical studies^13^ suggest that the imbalance of the *xz*/*yz* orbitals in the order of 10 meV resolves the magnetic frustration between (π, π) and (π, −π), resulting in the enhancement of spin fluctuations. Nuclear magnetic resonance studies^23^ on AsP122 reported the spin fluctuations evolving around *T*_o_ in a wide range of *x*, which possibly supports the above scenario.

For an optimally doped sample at *x* = 0.3, we observed the orbital anisotropy appearing below ∼90 K, which is not suppressed by the onset of superconductivity (Fig. 3). While the orthorhombic structural phase has not been reported from XRD measurements^24^ at any *T* for *x* = 0.30, the present ARPES study may suggest the existence of some form of orthorhombicity, e.g. the local variation or fluctuation of the orthorhombicity, as detected for BaFe_2_As_2_ by neutron powder diffraction^25^. Nevertheless, recent polarized ultrafast optical measurements^26^ revealed a two-fold symmetric optical response occurring approximately at 40–60 K for an optimally doped AsP122 (*x* = 0.31), which can be explained by the orbital anisotropy in the present ARPES study. Scanning Laue microdiffraction measurements^26^ further suggested that such electronic anisotropy was decoupled from the strain anisotropy on the crystal surface. These results imply that the orbital anisotropy in the present ARPESstudy may not necessarily correspond to the presence of the lattice distortion.

The orbital anisotropy observed by the ARPES can also be interpreted as a dynamical ferro-orbital order, when the photoexcitation process is much faster than the time scale of the dynamical fluctuations. In the case of FeSe^11^ and NaFeAs^8^, the orbital anisotropy rapidly increases below *T*_*s*_, which suggests an instantaneous evolution of the ferro-orbital order at the orthorhombic structural transition. Regarding AsP122, there is a possibility that the anomalous normal state is characterised by the dynamical ferro-orbital order fluctuation extended in a wide range of the phase diagram. Another possibility is that the orbital anisotropy observed in the present ARPES study indicates the presence of an antiferro-type orbital order^13^. A recent ARPES study^27^ on Ba,KFe_2_As_2_ (BaK122) suggested that an antiferroic electronic structure is realised in a large portion of the non-magnetic state including the SC phase. The antiferroic electronic instability in BaK122 does not compete with the onset of superconductivity similarly to that observed for the orbital anisotropy in AsP122 (Fig. 3). It should be noted that the *x–T* region where the orbital anisotropy appears overlaps with the pseudo-gap (PG) region reported by an ARPES experiment^7^ and optical measurements^28^. Theoretical studies^29,30^ predict that spin-nematicity induces the PG in the density of states significantly above *T*_s_. While the origin of the electronic nematicity in AsP122 needs to be further investigated, these results suggest that superconductivity in AsP122 emerges at the PG state where the *C*_4_ rotational symmetry is broken in lattice, magnetism, and orbital degrees of freedom. The different *x*- and *T*-dependences of the orbital anisotropy might be the key components for the systematic understanding of the diverse phase diagrams and SC gap symmetries in the iron-pnictide/chalcogenide superconductors.

In conclusion, we investigated the orbital anisotropy in the AsP122 system by using ARPES. We found that orbital anisotropy widely exists in the non-magnetic state, including the SC phase. It is monotonically suppressed toward the optimally doped regime and not observed around the over-doped regime. The orbital anisotropy, which possibly accompanies the anisotropic lattice and magnetism, is probably a crucial phenomenon to understand the anomalous normal state with PG and spin fluctuations, as realised in AsP122.

## Methods

### Sample growth and characterisation

Single crystals of AsP122 with *x* = 0.00 (*T*_N,s_ = 136 K), *x* = 0.07 (*T*_N,s_ = 114 K), *x* = 0.24 (*T*_N,s_ = 55 K, *T*_c_ = 16 K), *x* = 0.30 (*T*_c_ = 30 K), *x* = 0.45 (*T*_c_ = 22 K), *x* = 0.52 (*T*_c_ = 15 K), *x* = 0.61 (*T*_c_ = 9 K), *x* = 0.74, and *x* = 0.87 were grown by a self-flux method as described in refs^31^ and^32^. Resistivity measurements^32^ suggest the high quality of the crystals with a residual resistivity ratio of ≤30.

### Photoemission measurements

The ARPES measurements were performed using a VG Scienta R4000 WAL electron analyzer and a helium discharge lamp of *hν* = 40.8 eV. The energy resolution was set to 10 meV. The single crystals were cleaved at 200 K in ultrahigh vacuum of 5 × 10^−11^ Torr. The magnitude of the unidirectional strain applied by the detwinning device was in the order of 10 MPa^33^. The spectra were reproducible over 24-h measurement cycles. The *E*_*F*_ of the samples was referenced to that of a gold film evaporated onto the sample holder.
